# Fowler’s Syndrome—The Cause of Urinary Retention in Young Women, Often Forgotten, but Significant and Challenging to Treat

**DOI:** 10.3390/ijerph18063310

**Published:** 2021-03-23

**Authors:** Jacek K. Szymański, Aneta Słabuszewska-Jóźwiak, Grzegorz Jakiel

**Affiliations:** First Department of Obstetrics and Gynecology, Centre of Postgraduate Medical Education, Żelazna 90 Str., 01-004 Warsaw, Poland; anetaslabuszewska@gmail.com (A.S.-J.); grzegorz.jakiel1@o2.pl (G.J.)

**Keywords:** urinary retention, Fowler’s syndrome, sacral neuromodulation

## Abstract

Urinary retention in young women is a relatively rare clinical problem and is often underdiagnosed. In particular, functional causes of urinary retention pose a diagnostic challenge. One of them is Fowler’s syndrome, which is associated with impaired urethral relaxation. Fowler’s syndrome is characterized by a large bladder capacity, reduced sensation, increased maximal urethral closure pressure, and detrusor underactivity. Several hypotheses have arisen to explain the cause of urethral relaxation disorders: hormonal changes characteristic of Polycystic Ovary Syndrome (PCOS), causing abnormal stabilization of the muscle membrane, primary failure of relaxation of the striated muscle of the urethra sphincter, and increased urethral afferent activity, inhibiting the bladder afferent signals from reaching the brain by potentiating a spinal mechanism of urinary continence. Currently, sacral neuromodulation is the only intervention that can restore an atypical voiding pattern in women with Fowler’s syndrome. The therapeutic effectiveness exceeds 70%, although the revision rate is relatively high, exceeding 50%. Well-designed, long-term prospective studies comparing sacral neuromodulation (SNM) with other therapies such as pelvic floor muscle physiotherapy are warranted to offer the best patient-tailored treatment.

## 1. Introduction

Urinary retention in young women is a relatively rare clinical problem. Epidemiological studies estimate that its incidence in young women ranges from 3 cases per 100,000 per year to 0.3% after the exclusion of other causes, such as postoperative, postpartum, gynecological, urological, rectal, and psychiatric causes [1,2,3]. The incidence of Fowler’s syndrome is rated at 0.2 cases per 100,000 per year [4].

Urinary retention could be related to bladder outlet obstruction (BOO) or detrusor underactivity (DUA), or it may constitute a combination of these two causes. The underlying pathology could be associated with anatomical, neurogenic, and myogenic factors, or it may result from pharmacotherapy or functional reasons where no organic cause is identified. [5]. The anatomical (mechanical) factors include, among others, pelvic organ tumors, stenosis of the bladder neck, urethral diverticulum, pelvic organ prolapse, and previous pelvic surgery. The functional causes are a result of the pathological changes in the contraction of the periurethral muscles (dysfunctional voiding and detrusor sphincter dyssynergia) or impaired urethral relaxation (Fowler’s syndrome) [6]. Urinary retention caused by mechanical BOO is usually successfully treated with medication or surgery, whereas the management of urinary retention resulting from detrusor underactivity or functional BOO remains a challenge. However, urinary retention caused by DUA as a consequence of pharmacotherapy is usually transient and reversible [5]. This study aimed to review the literature on the current knowledge of Fowler’s syndrome with consideration of sacral neuromodulation as a modern therapeutic method.

## 2. Materials and Methods

A literature search was performed in August 2020 using the Medline and Embase databases, ranging from 1988 to February 2021. The adopted criteria allowed for the inclusion of studies on humans and animals, original papers, and prospective and retrospective trials limited to those published in English. Studies on the effectiveness of neuromodulation in the treatment of Fowler’s syndrome were identified using various medical subject headings. The search for relevant records was carried out using the following phrases: “Fowler’s syndrome”, “Fowler’s syndrome and sacral neuromodulation”, “urinary retention in women”, and “urinary retention in women and sacral neuromodulation”.

## 3. Results

**Figure 1:**
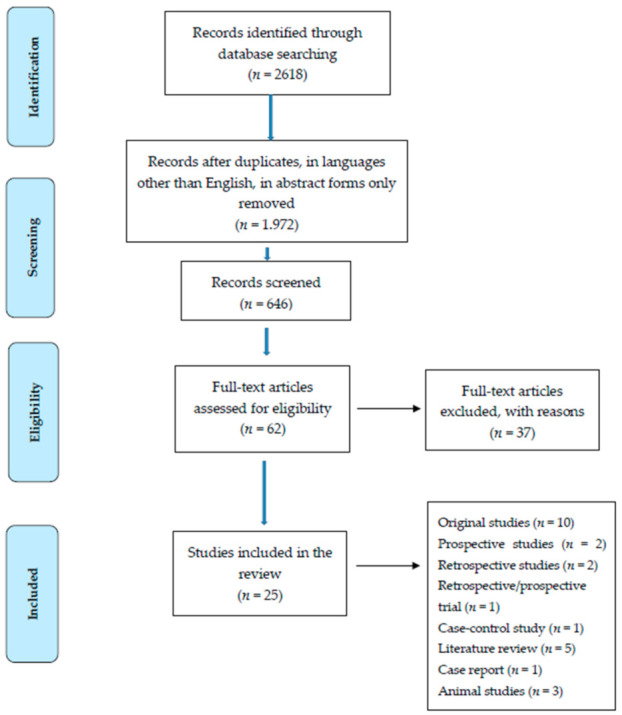
Flow chart presenting the process of searching for the eligible articles.

A total of 2618 articles were identified. Articles in languages other than English, duplicates, articles available only in the form of abstracts, and papers not closely related to the subject of this study were excluded. Ultimately, 25 articles were selected. This included ten original studies, two prospective studies, two retrospective studies, one retrospective and subsequently prospective trial, and one case–control study. Moreover, five literature reviews and one case report were included (Figure 1).

Three of the studies had been conducted on animals. The number of participants in the original studies varied and ranged from 6 to 87. In retrospective and prospective studies, the number of subjects ranged from 18 to 138, with a mean follow-up of 13.5 years (12–15 years) and 10.5 months (9–12 months), respectively. The relevant studies are listed in Table 1.

### 3.1. Clinical Picture of Fowler’s Syndrome

Fowler’s syndrome typically occurs in post-menarche young women in the second and third decades of life. Most of the patients reveal a trigger medical event in their history, such as gynecological surgery or other surgical procedures, childbirth, and acute medical conditions [5]. Trachta et al. reported a case of a 14-year-old girl who developed urinary retention after an uncomplicated laparoscopic appendectomy [7]. Furthermore, before the onset of retention, these women are likely to have had relatively mild voiding dysfunction, such as infrequent voiding or an intermittent stream [8]. Moreover, young women undergo various surgical procedures, such as urethral dilatation, urethrotomy, hysterectomy, myomectomy, and treatment of endometriosis, before a diagnosis of FS is established. As mentioned above, the prevalence of FS in the female population is rare; thus, an awareness of this pathology in the medical community is crucial for making a proper diagnosis. A detailed medical history that can exclude other possible causes of urinary retention is essential. Certain drug groups such as antidepressants, sedatives, and opiates are well-recognized causes of urinary retention [5,9]. Every young woman with urinary retention should undergo a full evaluation of the lower urinary tract with an assessment of neural control. The clinical features of Fowler’s syndrome are listed in Table 2.

### 3.2. Pathophysiology of Fowler’s Syndrome

The etiology of Fowler’s syndrome remains unclear. One of the contributing factors is possibly the specific hyperactivity of the external urethral sphincter, which can be diagnosed using a concentric needle (electromyography) EMG electrode. Detailed EMG analysis shows that there are two components to sphincter activity: complex repetitive discharges (CRDs) and decelerating bursts [10]. CRDs appear as the direct spread of electrical activity from one muscle to another. They do not originate from neuromuscular transmission but are transmitted via adjacent membranes in parallel waves [10]. In this way, the CRDs create autonomous circuitous excitatory activity, leading to impaired relaxation of the urethral sphincter during voiding [5]. On the other hand, abnormal EMG activity has been found in 30–53% of healthy, asymptomatic women [11,12]. It is difficult to quantify the “amount” of abnormal EMG activity due to the small diameter of the sphincter muscle fibers and the limitations of the measuring techniques. Based on the observation that women with urinary retention often demonstrate polycystic ovaries, Fowler linked the increased sphincter activity to a hormonal disorder. This hypothesis explains the impaired transmission of the electrical impulses throughout the muscle by the improper muscle membrane stability as a consequence of ion channel dysfunction (hormonal channelopathy). In another study, over 80% of patients with Fowler’s syndrome had one or more gynecological disorders. The most prevalent were endometriosis, polycystic ovarian syndrome (PCOS), and subfertility. Although the incidence of these pathologies reached statistical significance, their occurrence in the FS group and the controls stayed within the population range, making the theory of the increased concomitance of FS with other gynecological pathologies tenuous [13,14]. Another theory assumes that the primary failure of relaxation of the striated muscle of the urethral sphincter leads to a raised urethral pressure profile. An ultrasound study carried out by Noble et al. [15] in women with obstructed voiding and abnormal sphincter EMG activity found evidence of local muscle hypertrophy. These findings were not confirmed by endoscopic ultrasound biopsy, suggesting that increased sphincter volume could be caused by components other than the skeletal muscle [16].

**Figure 2:**
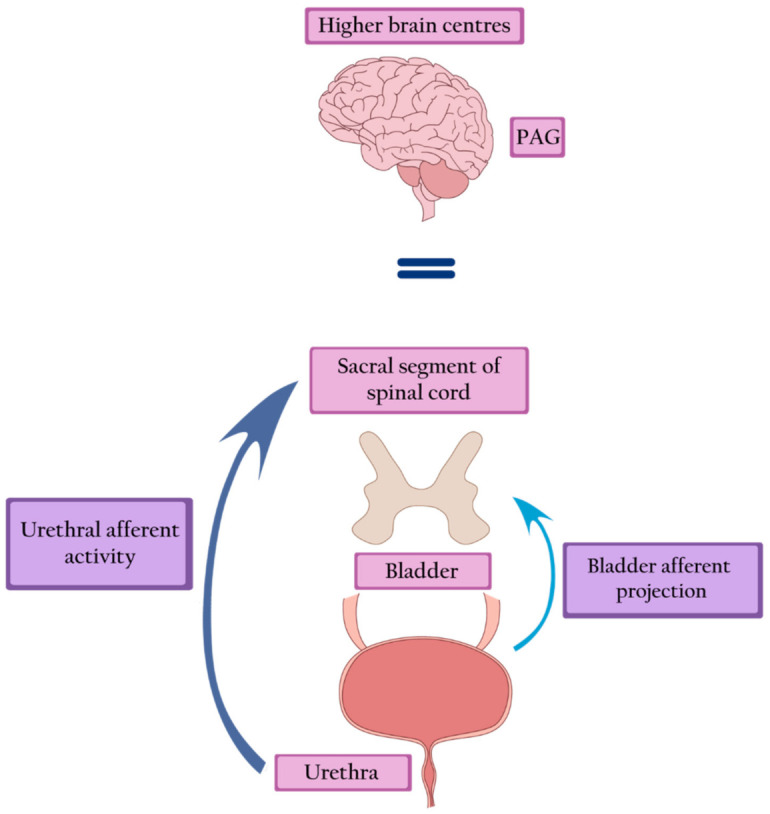
Fowler’s syndrome—strong afferent signals generated by the urethra inhibit the afferent activity of the bladder, leading to inactivation of the PAG and higher brain centers. PAG—periaqueductal grey.

Large bladder capacity, reduced sensation, increased maximal urethral closure pressure (MUCP), and detrusor underactivity constitute the urodynamic characteristics of Fowler’s syndrome [17]. Increased urethral afferent activity most likely inhibits the bladder afferent signals from reaching the brain by potentiating a spinal mechanism of urinary continence. During the filling phase, strong urethral afferent signals are generated. They reduce the signal transmission to the periaqueductal grey (PAG) and higher centers by inhibiting bladder afferent activity in the sacral cord. Thus, the increased activity of the striated urethral sphincter leads to halted voiding [18] (Figure 2).

### 3.3. Neuroimaging Studies

The hypothesis described above has been supported by two functional neuroimaging studies which have demonstrated improper brain activity in response to bladder filling in patients with Fowler’s syndrome. Dasgupta and colleagues used positron emission tomography (PET) in women with Fowler’s syndrome and healthy controls to identify the regions of brain activity that are responsible for the perception of bladder fulness and their modulation by sacral nerve stimulation [19]. They demonstrated that bladder fullness in healthy females is associated with enhanced activity within the brainstem (midbrain and pons) and the anterior and posterior cingulate cortices. Interaction between these structures is essential for the control of micturition. Brainstem regions provide the first level of supraspinal control of urinary function. The PAG receives afferent signals from the sacral spinal cord and thereby communicates with the efferent pontine micturition center. Subsequently, the pontine micturition center projects to the sacral parasympathetic preganglionic neurons.

Moreover, this brain imaging study revealed that the representation of bladder fullness is not solely localized to the PAG. Despite this, the activated area may be more diffused, involving other midbrain regions such as the substantia nigra. Neurons located in the substantia nigra and ventral tegmentum respond to bladder filling and support the micturition reflex. The PAG, the substantia nigra, and the ventral tegmental area are involved in a complicated network of brain centers. The anterior cingulate cortex (ACC) and posterior cingulate cortex (PCC) are essential parts of this network. The ACC likely supports the integration of visceral afferent information. It facilitates an autonomic and motor response through a combination of internal motivations and external behavioral signals. The posterior cingulate cortex (PCC) plays a complementary role in relation to bladder filling. It is suggested that the function of the ACC is primarily executive, whereas the PCC has been defined as more evaluative [20]. It is plausible that the PCC may prevent the bladder overfilling by supporting a motivational representation of bladder extension, leading to micturition.

In contrast to this study, other research utilizing functional magnetic resonance imaging (fMRI) showed widespread negative responses to bladder infusion in women with FS, which were different from activation in healthy individuals. At baseline, with an empty bladder, the brain response to bladder filling was predominantly negative. Furthermore, this negative response was enhanced by the increase in the maximum urethral closure pressure (MUCP), resulting in the deactivation of centers in the midbrain and cortex. These findings support the hypothesis that afferent signals from the bladder are inhibited in women with FS and never reach the PAG [18].

### 3.4. Animal Models

Recently published studies on the feline model have contributed to a more accurate understanding of the pathogenesis of nonobstructive urinary retention (NOUR). Li et al. [21] showed that the abnormal somatic stimulation of afferent axons in the tibial nerve leads to a NOUR that persists for more than 2 hours after the end of stimulation. This observation confirms that the pathophysiology of Fowler’s syndrome is associated with the tonic, afferent activation of the pudendal nerve arising in the external urethral sphincter (EUS). It is important to note that the modulatory effect of tibial nerve stimulation (TNS) is frequency-dependent. The urinary retention obtained by the 5-Hz TNS is partially reversed at 1-Hz TNS. Thus, the voiding reflex in the CNS can be inhibited or induced by modulating signals in the tibial afferents. To reflect the pathophysiology of Fowler’s syndrome more accurately, another animal model was created in which pudendal nerve stimulation (PNS) was performed. The persistence of the bladder underactivity depended on the time and intensity of the stimulation. Short-term PNS resulted in the rapid disappearance of bladder inactivity, while prolonged PNS at a higher intensity resulted in bladder inactivity lasting for 1.5–2 h after the cessation of stimulation. The study results confirmed the inhibitory effect of pudendal nerve stimulation on the micturition reflex. Moreover, it has been shown that not only tonic but also intermittent afferent activity can generate long-lasting inhibition of bladder contraction. Both PNS and TNS induce bladder inactivity, confirming that prolonged somatic afferent activity may be the pathophysiological cause of NOUR. The advantage of the presented animal models is the maintenance of neural control over the bladder, which makes them more compatible with the real idiopathic underactive bladder [22].

### 3.5. Treatment

**Figure 3:**
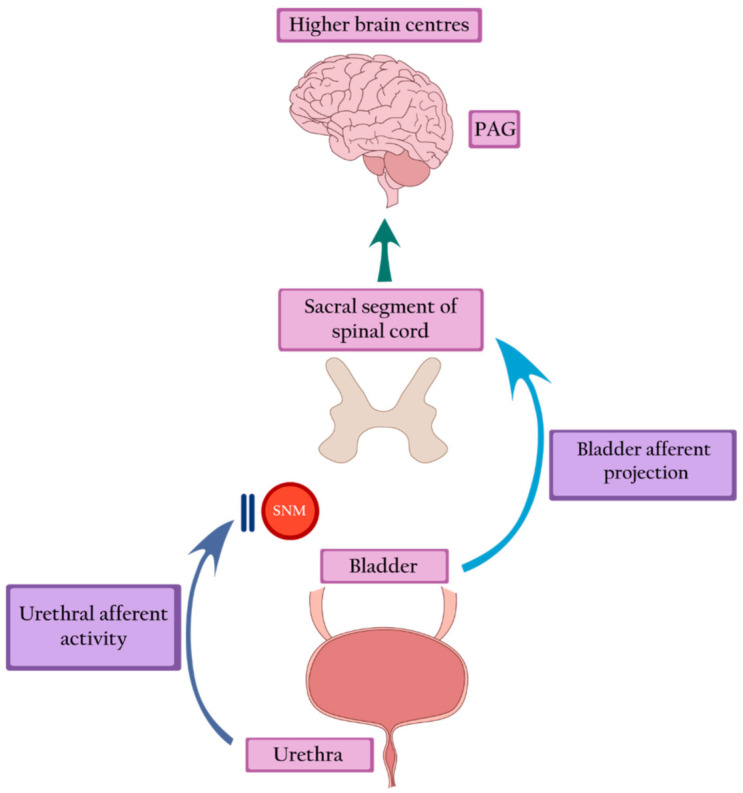
Sacral neuromodulation blocks urethral inhibition and restores bladder afferent projection, leading to the return of bladder sensation and activation of the PAG. PAG—periaqueductal grey, SNM—sacral neuromodulation.

Sacral neuromodulation is defined as the permanent electrical stimulation of the sacral segment of the spinal cord that controls the functioning of the bladder and pelvic floor. It restores the balance in the transmission of signals between the pelvic organs, the spinal cord, and the higher centers of the central nervous system [23]. Sacral neuromodulation is the only intervention that can restore a typical voiding pattern in women with Fowler’s syndrome. Brain imaging studies show the restoration of the midbrain and pontine activity, as well as the normalization of the signal transfer between the brainstem and the cortex. Moreover, SNM attenuates anterior cingulate activity, thus restoring the desire and ability to void [19]. Presumably, by stimulating neurons in the spinal cords, SNM blocks the inhibition of spinal information transfer from the bladder [18] (Figure 3)**.**

However, the urodynamic evaluation of SNM therapy raises questions concerning a direct afferent effect. Although SNM restores normal voiding, it does not have a direct relaxant effect on the EUS. No changes have been demonstrated in EMG and MUCP before and after implantation (MUCP 92.9 cm H_2_O pre-SNM vs. 84.9 after stimulation, *p* = 0.06). As the abnormality persists, many of the patients void in an obstructed manner. Uroflowmetry shows an interrupted stream. Surprisingly, cystometry fails to reveal significantly elevated detrusor pressure, as could be expected due to a chronically unrelaxed sphincter. A modest increase in detrusor pressure is most likely sufficient in overcoming urethral resistance. The impact of SNM on detrusor contraction is consistent with its inhibiting effect on urethral afferent activation [17]. This hypothesis was confirmed by an animal study which showed that sacral neuromodulation could partially suppress or completely block the increase in bladder capacity induced by pudendal nerve stimulation [24]. In this context, voiding restoration seems to be achieved through a combination of spinal and supraspinal mechanisms. The clinical efficacy of SNM in the treatment of urinary retention was confirmed in several trials. Swinn et al. (2000) showed a 68% success rate, yet a relatively high reoperation rate of 21% [25]. Another study demonstrated the efficacy of SNM in 77% of patients at a 5-year follow-up. Shortly after implantation, 96% of subjects (25 patients) voided. However, two had their stimulator deactivated within five years due to pregnancy, and a further three reported loss of efficacy. The revision rate was 54%, and the most common complications included loss of effectiveness, implant-related discomfort, and leg pain [17]. The next study reported twelve years of experience in SNM therapy offered to the women with nonobstructive urinary retention, based on 27 individuals with a median follow-up of 5.7 years. A success rate of 70.83% was reflected in the return of spontaneous voiding and reduction of mean postvoid residual (PVR) urine from 402.2 to 28.1 mL (*p* < 0.0001) [26]. Some hopes in the treatment of Fowler’s syndrome have been linked to the injection of botulinum toxin A (BoNT-A) into the external urethral sphincter as a less invasive procedure than SNM. The first transperitoneal 200-U BoNT-A injections performed by Fowler in 1992 were unsuccessful [27]. In the following years, transurethral or periurethral injections of 50–100 U BoNT-A were attempted several times, resulting in an improvement of 37%–43% of female patients. The limitations of the trials were the small size of the study groups, the lack of control groups, various injection techniques, and different doses of BoNT-A [28].

## 4. Discussion

In 1988, Professor Clare J. Fowler and her colleagues described a syndrome in young women who demonstrated urinary retention as the primary symptom, accompanied by an abnormality in electromyography (EMG) of the striated muscle of the urethral sphincter, which was related to a pathological contraction of the muscle [13,29]. As this problem is rare, epidemiological data on Fowler’s syndrome are scarce. Fowler’s syndrome is challenging to diagnose and, therefore, this pathology can often be overlooked. The rarity of Fowler’s syndrome limits the organization of extensive clinical trials in order to thoroughly understand its pathophysiology and select the best therapeutic method. There are no prospective, randomized, and controlled trials with a long follow-up period or in-depth diagnostics that would exclude other causes of urinary retention. Multi-annual observations relate only to retrospective studies. Brain imaging studies and well-designed animal research provide a better understanding of the etiology of NOUR. Based on these studies, it seems that the main therapeutic direction should be interference with the bidirectional transmission of external urethral sphincter-central nervous system nerve impulses, in order to reverse or inhibit pathological excitation and reflexes. The role of neurotransmitters should also be considered in future therapeutic options; for example, one hypothesis suggests the upregulation of spinal cord enkephalins in Fowler’s syndrome. It is possible that SNM acts through neurotransmitters with an anti-enkephalin effect [9]. The recently published studies on the animal models cited above have demonstrated a long-term inhibitory effect on bladder contractility in the function of the intensity and duration of TNS/PNS. On one hand, such a model makes it possible to conduct further research in order to better understand the pathophysiology of NOUR and to develop further therapeutic options; on the other hand, it seems possible to use this model to optimize and individualize SNM therapy in patients with an overactive bladder.

The exact mechanism of action of SNM is not fully understood. However, it seems that due to its high efficiency, it is currently the best therapeutic option to avoid or reduce the number of catheterizations in women with NOUR. SNM has a number of limitations related to the occurrence of complications, such as undesirable change in stimulation, implant site pain, lead migration, therapeutic ineffectiveness, and a lower libido. The revision rate exceeding 50%, caused by side effects, results in the necessity to remove the implant in over 20% of patients [30,31,32,33]. Further limitations of the method include absolute contraindications to its use—including an inadequate clinical response to a therapeutic trial, lack of efficient supportive care, and pregnancy—and relative contraindications, such as established complete spinal cord injury, a severe or rapidly progressive neurological disease, and abnormal sacral anatomy [34]. After all, the high cost of the therapy, reaching GBP 10,000, is not without significance [35]. However, in a Canadian study, the cost-effectiveness of SNM was higher when compared to BoNT-A injections over a 10-year time horizon [36]. The future of neuromodulation is tempting. Miniaturized, rechargeable devices that are safe during full-body MRI scans are being introduced to the market [37]. It is possible that, in the future, wireless devices generating kHz frequencies capable of blocking pudendal nerve conduction will also be used in the treatment of detrusor sphincter dyssynergia (DSD) in people with spinal cord injuries [38].

## 5. Conclusions

Urinary retention (including Fowler’s syndrome) in young women is complex, poorly understood, and often underdiagnosed due to its rarity. The medical community should be aware of this pathology disrupting the quality of life of young women. Further research should be undertaken to clarify the etiology of FS, as well as to improve and simplify the diagnostic process. Nowadays, SNM is the only therapy that effectively restores voiding in the majority of patients, most likely by reestablishing afferent transmission to the brain. Nevertheless, well-designed, long-term prospective studies comparing SNM with other therapies such as pelvic floor muscle physiotherapy are warranted to offer the best patient-tailored treatment.

## Figures and Tables

**Table 1:** Original studies on sacral neuromodulation. PCOS—Polycystic Ovary Syndrome, CRDs—complex repetitive discharges, DBs—decelerating bursts, EUS—external urethral sphincter, TRUS—transrectal ultrasonography, SNM—sacral neuromodulation, FS—Fowler’s syndrome, PAG—periaqueductal grey, DRS—dorsal root stimulation, INUR—idiopathic nonobstructive urinary retention, TNS—tibial nerve stimulation, PNS—pudendal nerve stimulation.

Author	Type of Study	Aim	Primary Outcome
Fowler et al., 1988	Cross-sectional	To reveal the cause of unexplained urinary retention in young women	Abnormal electromyographic activity of the external urethral sphincter in women with urinary retention associated with PCOS
Fowler et al., 1985	Observational	To elucidate the electromyotonic activity of the external urethral sphincter in women with urinary retention	Complex repetitive discharges and decelerating bursts, ephaptic spread of excitation between muscle fibers
Tawadros et al., 2015	Cross-sectional	To investigate the presence of CRDs and DBs in the EUS during the menstrual cycle in females with no urinary symptoms	CRDs and DBs in the EUS are present in 53% of asymptomatic women, more commonly in the luteal phase of the cycle
Ramm et al., 2012	Cross-sectional	To compare the proportion of women with CRDs among females with and without urinary disorders	CRDs detected in 30% of asymptomatic women
Noble et al., 1995	Cross-sectional	TRUS assessments of the EUS volume in women with obstructed voiding and abnormal electromyography (EMG) activity and in asymptomatic women	The volume of the EUS in the control group was significantly lower than in the obstructed group
Andrich et al., 2005	Cross-sectional	Morphological description of the urethral rhabdosphincter in women with and without urinary retention	No difference in rhabdosphincter fiber diameter was found in women with urinary retention and control
DasGupta, Fowler 2004	Cross-sectional	SNM in restoring voiding function in women with urinary retention	SNM has no direct relaxant effect on the sphincter
Kavia et al., 2010	Etiology (case series)	To examine brain responses to bladder filling in women with FS treated with SNM	Overactive urethra inhibits afferent signals blocking bladder afferent activity at the sacral level, deactivates the periaqueductal grey (PAG) and higher center, causing a loss of bladder sensation. SNM blocks inhibition by urethral afferents at the sacral level
DasGupta et al., 2005	Etiology (cross-sectional)	To investigate how SNM acts on brain centers involved in bladder function in women with FS and control	Enhanced limbic cortical activity with no significant activity in the brainstem was detected while bladder fulness was present in women with FS. SNM restores the midbrain activity and decreases cortical activity in this group
Karmarkar et al., 2015	Case–control study	To assess the prevalence of gynecological pathologies in women with FS	Statistically significant relationship between FS and endometriosis (*p* = 0.003), and FS and PCOS (*p* = 0.003), was found
Panicker et al.,2012	Observation from Prospective Clinical Study	To identify the impact of opiates on urinary retention in women	Exogenous opiates may compound any functional abnormalities predisposing women to urinary retention
Li et al., 2018	Animal study	To test the hypothesis that DRS blocks pudendal afferent inhibition of the micturition reflex	DRS blocks pudendal afferent inhibition and restores bladder capacity to control level
Swinn et al., 2000	Case–control study	To evaluate the efficacy of SNM in the treatment of FS	Success rate 68% Reoperation rate 21%
Mehmood et al., 2017	Retrospective study	To determine the safety and efficacy of SNM in the treatment of INUR	Improvement rate 83.3% Reoperation rate 41.6%
Li et al., 2020	Animal study	To determine the effects of TNS on reflex bladder activity	Repeated application of TNS produced long-lasting bladder underactivity
Mohapatra et al., 2021	Animal study	To determine the effects of PNS on reflex bladder activity	Repeated application of PNS resulted in long-lasting bladder underactivity

**Table 2:** Clinical features of Fowler’s syndrome [5].

Examination	Feature
History	Young women, post-menarche, 2nd to 3rd decades Triggering event (surgery, acute illness) Variable association with PCOS Painless retention with a large residual volume of urine (> 1000 mL) Pain or difficulty most commonly when removing the catheter
Urological, gynecological, and neurological assessment	No identified structural or neurological cause of urinary retention
Urodynamic studies	Large bladder capacity Decreased bladder sensation Reduced or absent detrusor contraction Reduced or absent flow Open bladder neck with narrowing in the midurethra/ballooning of the proximal urethra
Concentric needle urethral sphincter electromyography	Complex repetitive discharges and decelerating bursts
Trans-vaginal sphincter ultrasound	Increased sphincter volume
Urethral pressure profilometry	Increased maximal urethral closure pressure

## Data Availability

Not Applicable.
